# Association of *PTPRD/PTPRT* Mutation With Better Clinical Outcomes in NSCLC Patients Treated With Immune Checkpoint Blockades

**DOI:** 10.3389/fonc.2021.650122

**Published:** 2021-05-27

**Authors:** Xiaoyan Wang, Bingchen Wu, Zhengqing Yan, Guoqiang Wang, Shiqing Chen, Jian Zeng, Feng Tao, Bichun Xu, Honggang Ke, Mei Li

**Affiliations:** ^1^ Department of Respiratory Medicine, Affiliated Hospital of Nantong University, Nantong, China; ^2^ Department of Oncology, Hospital of Chinese Medicine of Changxing County, Huzhou, China; ^3^ The Medical Department, 3D Medicines Inc., Shanghai, China; ^4^ Department of Thoracic Surgery, Zhejiang Cancer Hospital, University of Chinese Academy of Sciences, Hangzhou, China; ^5^ Department of Respiratory Medicine, The Affiliated Hospital of Jiaxing University, Jiaxing, China; ^6^ Department of Radiotherapy, Second Affiliated Hospital of Soochow University, Suzhou, China; ^7^ Department of Cardiothoracic Surgery, Affiliated Hospital of Nantong University, Nantong, China; ^8^ Department of Medical Oncology, Affiliated Hospital of Nantong University, Nantong, China

**Keywords:** *PTPRD*, *PTPRT*, JAK-STAT, immune checkpoint blockades, non-small cell lung cancer

## Abstract

The common gamma receptor–dependent cytokines and their JAK-STAT pathways play important roles in T cell immunity and have been demonstrated to be related with response to immune checkpoint blockades (ICBs). PTPRD and PTPRT are phosphatases involved in JAK-STAT pathway. However, their clinical significance for non-small cell lung cancer (NSCLC) treated with ICBs is still unclear. Genomic and survival data of NSCLC patients administrated with anti–PD-1/PD-L1 or anti–CTLA-4 antibodies (Rizvi2015; Hellmann2018; Rizvi2018 Samstein2019) were retrieved from publicly accessible data. Genomic, survival and mRNA data of 1007 patients with NSCLC were obtained from The Cancer Genome Atlas (TCGA). *PTPRD/PTPRT* mutation was significantly associated with better progression-free survival (PFS) in three independent Rizvi2015, Hellmann2018 and Rizvi2018 cohorts. The median PFS for *PTPRD/PTPRT* mutant-type *vs*. wild-type NSCLC patients were not reached *vs*. 6.3 months (Rizvi2015, HR = 0.16; 95% CI, 0.02-1.17; P=0.03), 24.0 *vs*. 5.4 months (Hellmann2018, HR, 0.49; 95% CI, 0.26-0.94; P=0.03), 5.6 *vs*. 3.0 months (Rizvi2018, HR = 0.64; 95% CI, 0.44-0.92; P=0.01) and 6.8 *vs*. 3.5 months (Pooled cohort, HR, 0.54; 95% CI, 0.39-0.73; P<0.0001) respectively. *PTPRD/PTPRT* mutation was an independent predictive factor for PFS in pooled cohort (P = 0.01). Additionally, *PTPRD/PTPRT* mutation associated with better overall survival (OS) in Samstein2019 cohort (19 *vs*. 10 months, P=0.03). While similar clinical benefits were not observed in patients without ICBs treatment (TCGA cohort, P=0.78). In the further exploratory analysis, *PTPRD/PTPRT* mutation was significantly associated with increased tumor mutation burden and higher mRNA expression of JAK1 and STAT1. Gene Set Enrichment Analysis revealed prominent enrichment of signatures related to antigen processing and presentation in patients with *PTPRD/PTPRT* mutation. This work suggested that *PTPRD/PTPRT* mutation might be a potential positive predictor for ICBs in NSCLC. These results need to be further confirmed in future.

## Introduction

Lung cancer is the leading cause of cancer death worldwide with 1.6 million deaths per year (1). Approximately 85% of cases are non-small cell lung cancer (NSCLC), of which lung adenocarcinoma (LUAD) and lung squamous cell carcinoma (LUSC) are the common histological subtypes (2). With development of molecular diagnosis, tyrosine kinase inhibitors (TKIs) have become the standard therapy for NSCLC patients harboring EGFR or ALK alterations over the past two decades and brought great clinical benefit for NSCLC patients. However, as for patients without driver oncogenic gene, the improvement in survival was minimal before the appearance of immune checkpoint blockades (ICBs).

ICBs have demonstrated significant clinical benefit in NSCLC patients, including antibodies targeting programmed death receptor-1 (PD-1), its ligand (PD-L1) and cytotoxic T-lymphocyte antigen-4 (CTLA-4). Unfortunately, only a subset of patients could respond to current immunotherapy strategies. In order to increase the response rate to ICBs, identifying the patients who can benefit from ICBs and developing novel potential strategies are two common methods. Encouragingly, several biomarkers have been proposed as distinct positive predictor for ICBs therapy, such as MSI-H, PD-L1 expression (3, 4), tumor mutation burden (TMB) (5, 6), and the intensity of CD8+ T cell infiltrates (7). Additionally, several genomic alterations had been found to be correlated with the clinical outcomes in NSCLC patients who received ICBs. Dong et al. reported that *TP53* and *KRAS* mutations in NSCLC were associated with the increased PD-L1 expression and activated T-effector and interferon-γ signature (8). Zhang et al. uncovered significant correlation between *North1/2/3* mutation and better efficacy of ICBs (9). On the contrary, some negative predictors for ICBs therapy, including *JAK1/2*, *MDM2/4* and *EGFR* alternations were also reported in previous works (10, 11). Currently, exploring the role of gene alternations in the NSCLC patients who received ICBs remains valuable for precision therapies.

Common gamma receptor–dependent cytokines and their JAK-STAT pathways play important roles in T cell immunity (12). It was reported that IFNγ/STAT1/STAT3 signaling axis related to the upregulation expression of PD-L1 in lung tumors (13). Additionally, STAT1 activation could trigger IRF-1 expression and subsequent initiated MHC class I antigen presentation-associated gene expression (14). Noted that protein tyrosine phosphatase receptor type D or T (PTPRD or PTPRT) are two of receptor-protein tyrosine phosphatases (R-PTPs) in NSCLC, which were reported as the mediator of JAK-STAT signal pathway (15, 16). However, to our best knowledge, the clinical significance of *PTPRD* and *PTPRT* alterations for NSCLC treated with ICBs is still unclear. In the present work, we aimed to explore the relationship between *PTPRD/PTPRT* mutation and clinical outcomes of ICBs in NSCLC patients.

## Materials and Methods

### Patients

Genomic and clinical data of NSCLC patients administrated with anti–PD-(L)1 or anti–CTLA-4 antibodies [Rizvi2015 (17), Hellmann2018 (18), Rizvi2018 (19) and Samstein 2019 (6)] were retrieved from publicly accessible data. The genomic, survival and mRNA data of 1226 patients with NSCLC were obtained from TCGA (www.cbioportal.org). As for the 3Dmed_NSCLC cohort, 1224 NSCLC patients were included from 22^nd^ October 2019 to 15^th^ April 2020 to explore the *PTPRD* and *PTPRT* mutation profiles in Chinese population. Their pathological diagnosis of the specimens was confirmed by hematoxylin and eosin (H&E) staining and the tumor tissue suffered to 733 cancer gene panel sequencing (3D Medicines Inc., Shanghai). All human sample collection and usage were in accordance with the principles of the Declaration of Helsinki and approved by Affiliated Hospital of Nantong University. All participated patients provided written consents.

### Study Design

Any mutation including nonsense, frameshift and missense mutation in *PTPRD* or *PTPRT* was defined as *PTPRD/PTPRT* mutation. *PTPRD/PTPRT* wild-type suggested that both *PTPRD* and *PTPRT* were wild-type. TMB-high group was defined as TMB ≥ median. The primary outcome was progression-free survival (PFS), which was calculated from the date of first immunotherapy administration to disease progression. The secondary outcome was overall survival (OS), which was calculated from the date of first immunotherapy administrated until death due to any cause. We explored the association between *PTPRD*/PRPRT mutation and PFS or OS using univariable and multivariable regression analysis.

### Statistical Analyses

Data analyses were performed using SPSS 20.0 (SPSS Inc., Chicago, IL). Survival description was illustrated by the Kaplan-Meier curves with P value determined by a log-rank test. Hazard’s ratio (HR) was determined through a cox proportional hazards regression model. The associations between PFS and various variables were examined by univariable and multivariable regression analysis. Continuous variables were compared by Mann-Whitney U test. False discovery rate (FDR) was used to adjust mRNA expression. Gene set enrichment analysis (GSEA) was used to determine potentially relevant gene expression signatures between patients harboring mutant-type or wild-type *PTPRD/PTPRT*. The java GSEA Desktop Application (GSEA 4.0.1) was downloaded from http://software.broadinstitute.org/gsea/index.jsp. The normalized enrichment score (NES) is the primary statistic for examining gene set enrichment results. The nominal P value estimates the statistical significance of the enrichment score. All reported P values were two-tailed, and P <0.05 or FDR <0.05 is considered statistically significant.

## Results

### Association Between *PTPRD*/*PTPRT* Mutation and Better PFS in NSCLC Patients Who Received ICBs Therapy From Three Independent Cohorts

The detailed baseline characteristics of NSCLC patients in three independent cohorts (Rizvi2015; Hellmann2018 and Rizvi2018) were summarized in **Table 1**. (1) The Rizvi2015 cohort contains 34 advanced NSCLC patients and their tumor tissues were subjected to whole-exome sequencing (WES). (2) The Hellmann2018 cohort contains 75 patients with NSCLC as part of the CheckMate-012 study and WES was performed on tumor tissues. (3) The Rizvi2018 cohort contains 240 patients with advanced NSCLC and their tumor tissues were profiled with MSK-IMPACT gene panels (341-gene, 410-gene or 468-gene panel). All the three gene panels included *PTPRD* and *PTPRT* genes. In the pooled cohort, 349 advanced NSCLC patients were included. The overall frequency of *PTPRD/PTPRT* mutation in Rizvi2015, Hellmann2018, Rizvi2018 and pooled cohorts were 15%, 31%, 20% and 21% respectively.

**Table 1:** Baseline characteristics of NSCLC patients in Rizvi2015, Hellmann2018, Rizvi2018 and pooled cohorts.

Characteristics	Rizvi2015	Hellmann2018	Rizvi2018	Pooled Cohort
Total n	34	75	240	349
Age, median (range)	62.5 (41-80)	66 (42-87)	66 (22-92)	65 (22-92)
Sex				
Male	16 (47%)	37 (49%)	118 (49%)	171 (49%)
Female	18 (53%)	38 (51%)	122 (51%)	178 (51%)
Cancer type n (%)				
Adenocarcinoma	34 (100%)	59 (79%)	186 (78%)	279 (80%)
Squamous	0 (0%)	16 (21%)	34 (14%)	50 (14%)
Others	0 (0%)	0 (%)	20 (8%)	20 (6%)
Agent				
PD-(L)1	34 (100%)	0 (0%)	206(86%)	240 (0%)
PD-(L)1+CTLA-4	0 (0%)	75 (100%)	34(14%)	109 (100%)
Smoking history, n (%)				
Current/former	28 (82%)	60 (80%)	193 (80%)	281 (80%)
Never	6 (18%)	15 (20%)	47 (20%)	68 (20%)
PDL1_expression				
≥1%	24 (70%)	45 (60%)	43 (18%)	112 (32%)
0%	6 (18%)	25 (33%)	41 (17%)	72 (21%)
Unknown	4 (12%)	5 (7%)	156 (65%)	165 (47%)
Gene, n (%)				
*PTPRD* mutation	3 (9%)	16 (21%)	30 (13%)	49 (14%)
*PTPRT* mutation	2 (6%)	9 (12%)	23 (10%)	34 (10%)
*PTPRD/PTPRT* mutation	5 (15%)	22 (29%)	47 (20%)	74 (21%)
*PTPRD/PTPRT* wild-type	29 (85%)	53 (71%)	193 (80%)	275 (79%)

**Figure 1:**
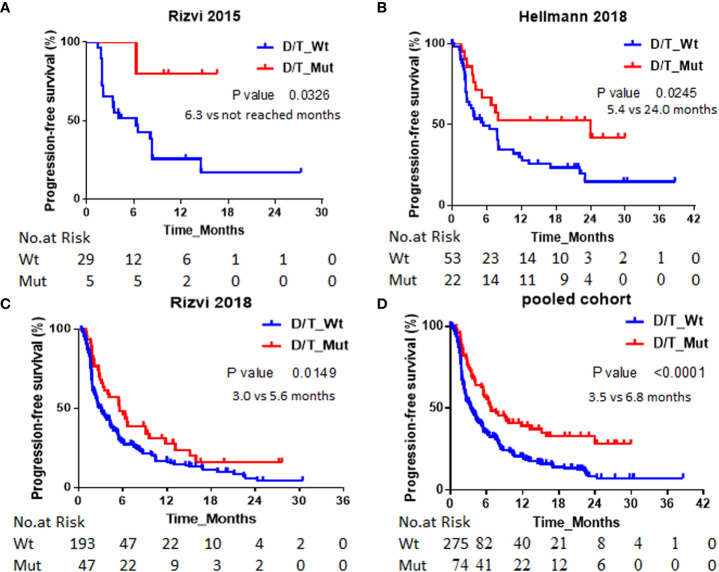
Association between *PTPRD/PTPRT* mutation and PFS. Kaplan-Meier survival curves of PFS comparing NSCLC patients with *PTPRD/PTPRT* mutant-type and wild-type in of **(A)** Rizvi2015, **(B)** Hellmann2018, **(C)** Rizvi2018 and **(D)** pooled cohorts respectively. *PTPRD/PTPRT* mutation: D/T_Mut; *PTPRD/PTPRT* wild-type: D/T_Wt.

The association between *PTPRD/PTPRT* mutation and PFS was analyzed in Rizvi2015, Hellmann2018, Rizvi2018 and pooled cohort respectively. As shown in **Figure 1**, *PTPRD/PTPRT* mutation was significantly associated with better PFS. The median PFS for *PTPRD/PTPRT* mutant-type *vs*. wild-type NSCLC patients were not reached *vs*. 6.3 months in Rizvi2015 cohort (HR = 0.16; 95% CI, 0.02-1.17; P=0.03), 24 *vs*. 5.4 months (HR, 0.49; 95% CI, 0.26-0.94; P=0.03) in Hellmann2018 cohort, 5.6 *vs*. 3.0 months (HR = 0.64; 95% CI, 0.44-0.92; P=0.01) in Rizvi2018 cohort and 6.8 *vs*. 3.5 months (HR, 0.54; 95% CI, 0.39-0.73; P<0.0001) in the pooled cohort respectively.

**Figure 2:**
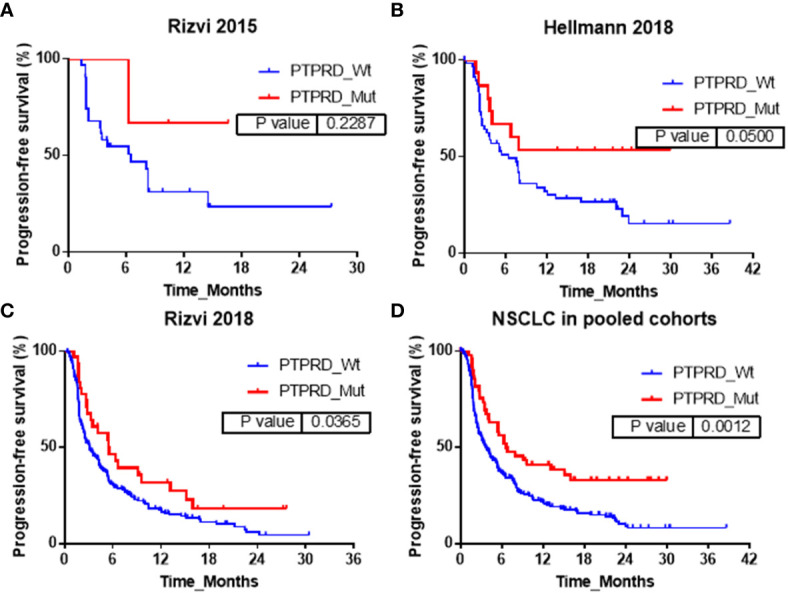
Association between *PTPRD* mutation and PFS in **(A)** Rizvi2015, **(B)** Hellmann2018, **(C)** Rizvi2018 and **(D)** pooled cohorts.

The association between *PTPRD* mutation and PFS was also explored in Rizvi2015, Hellmann2018, Rizvi2018 and pooled cohorts respectively. The corresponding results were shown in **Figure 2** respectively. In Rizvi2015 cohort, the median PFS in *PTPRD* mutant-type *vs*. wild-type NSCLC patients were not reached *vs*. 6.5 months (P=0.23). Although statistical significance was not obtained, *PTPRD* mutation tend to achieve longer PFS. In Hellmann2018 cohort, similar results also were observed, but with a marginal statistical significance. The median PFS in *PTPRD* mutant-type *vs*. wild-type NSCLC patients were not reached *vs*. 6.5 months (P=0.05). In Rizvi2018 cohort, *PTPRD* mutation were significantly associated with better PFS, and the median PFS in *PTPRD* mutant-type *vs*. wild-type NSCLC patients were 5.6 months *vs*. 3.1 months (P=0.04). In pooled cohort, significant association between *PTPRD* mutation and better PFS was also observed. The median PFS in *PTPRD* mutant-type *vs*. wild-type NSCLC patients were 6.6 months *vs*. 3.6 months (P=0.001).

**Figure 3:**
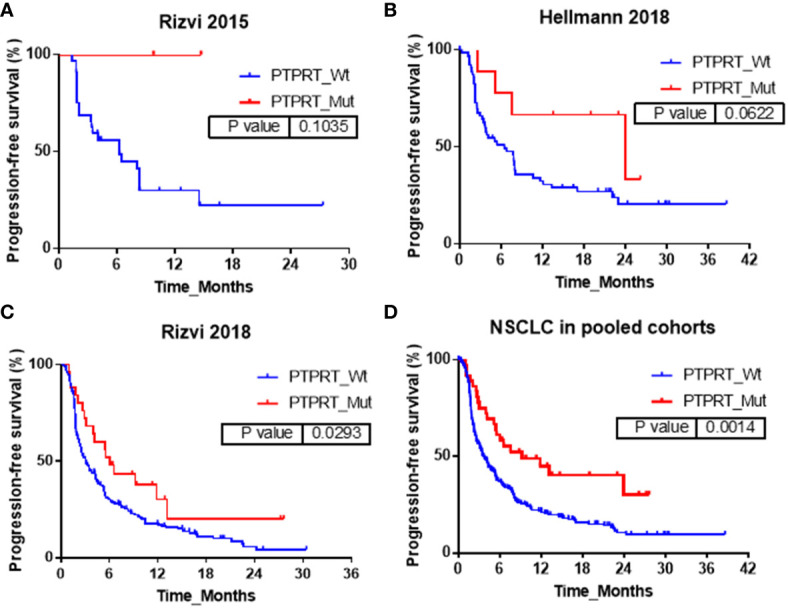
Association between *PTPRT* mutation and PFS in **(A)** Rizvi2015, **(B)** Hellmann2018, **(C)** Rizvi2018 and **(D)** pooled cohorts.

In addition, the influences of *PTPRT* mutation on PFS were also explored in Rizvi2015, Hellmann2018, Rizvi2018 and pooled cohorts respectively and the corresponding results were shown in **Figure 3**. In Rizvi2015 cohort, the median PFS in *PTPRT* mutant-type *vs*. wild-type NSCLC patients were not reached *vs*. 6.3 months respectively, with no statistically significant difference (P=0.10). In Hellmann2018 cohort similar results were also observed, but with a marginal statistical significance. The median PFS in *PTPRT* mutant-type *vs*. wild-type NSCLC patients were 24.0 months *vs*. 6.5 months (P=0.06). In Rizvi2018 cohort, *PTPRT* mutations were significantly associated with better PFS. The median PFS in *PTPRT* mutant-type *vs*. wild-type NSCLC patients was 6.0 months *vs*. 3.1 months (P=0.03). In pooled cohort, a significant association between *PTPRT* mutation and better PFS was also observed. The median PFS in *PTPRT* mutant-type *vs*. wild-type NSCLC patients were 9.2 months *vs*. 3.6 months (P=0.001).

The univariable and multivariable regression analysis of PFS the in Rizvi2015, Hellmann2018 and Rizvi2018 cohorts were summarized in **Table 2**. Several confounding factors were analyzed, including the age, sex and lines of therapy, PD-L1, TMB, smoker status, and *PTPRD/PTPRT* mutation. The multivariable analysis showed that *PTPRD/PTPRT* mutation was not significant associated with PFS in Rizvi2015 and Hellmann2018 cohort (HR 0.23, 95% CI, 0.03-2.04, p=0.18 and HR 0.59, 95% CI, 0.24-1.47, p=0.25, respectively). However, the multivariable analysis suggested that *PTPRD/PTPRT* mutation was an independent predictive factor for PFS in Rizvi2018 cohort (0.43, 95% CI, 0.20-0.92, p=0.03). The different outcomes between these independent cohorts might be due to the different size of patients. The deviation might exist in the conclusion of small cohorts (Rizvi2015, N=34 and Hellmann2018, N=75). In contrast, 240 NSCLC was included in the Rizvi2018 cohort. To further explore the influence of confounding factors on PFS, the univariable and multivariable regression analysis of PFS were also performed in pooled cohort. Notably, in the pooled cohort, the age (≥65 *vs*. <65 y), sex (male *vs*. female) and lines of therapy (lines of therapy ≥3 *vs*. <3), were not associated with PFS, no matter the univariable or multivariable regression analysis. In contrast, PD-L1, TMB, smoker status and *PTPRD/PTPRT* mutation were significantly associated with better PFS benefits. In the univariable analysis, PD-L1 status ≥1% *vs*. <1%, HR 0.60, 95% CI, 0.43-0.84, P =0.003; TMB≥ median *vs*. <median, HR 0.56, 95% CI, 0.44-0.72, P <0.001; Current or former *vs*. never smoker, HR 0.70, 95% CI, 0.52-0.93; P =0.01; *PTPRD/PTPRT* mutant-type *vs*. wild-type, HR 0.54, 95% CI, 0.39-0.73, p <0.001. In the multivariable analysis, PD-L1 status ≥1% *vs*. <1%, HR 0.67, 95% CI, 0.47-0.96, P =0.03; TMB≥ median *vs*. <median, HR 0.62, 95% CI, 0.42-0.92, P =0.02; Current or former *vs*. never smoker, HR 0.63, 95% CI, 0.41-0.97; P =0.04; *PTPRD/PTPRT* mutant-type *vs*. wild-type, HR 0.52, 95% CI, 0.31-0.87, p=0.01.

**Table 2:** Univariable and multivariable analyses of progression-free survival.

Rizvi2015				
Parameter	Univariable Analysis		Multivariable Analysis	
	HR (95%CI)	P value	HR (95%CI)	P value
Age ≥65 *vs*. <65 y	0.82 (0.33-2.01)	0.66	1.05 (0.33-3.38)	0.93
Male *vs*. female	1.75 (0.75-4.07)	0.20	1.09 (0.36-3.36)	0.88
Current or former *vs*. never smoker	0.60 (0.22-1.64)	0.32	0.67 (0.16-2.91)	0.60
TMB≥median *vs*. <median	0.21 (0.08-0.55)	0.002	0.20 (0.06-0.67)	0.01
PD-L1 status ≥1% *vs*. <1%	0.38 (0.14-1.01)	0.05	0.98 (0.33-2.98)	0.98
*PTPRD/PTPRT* mutant *vs*. wild	0.16 (0.02-1.17)	0.07	0.23 (0.03-2.04)	0.18
Lines of therapy ≥3 *vs*. <3	1.24 (0.53-2.91)	0.62	1.79 (0.46-6.93)	0.40
**Hellmann2018**				
**Parameter**	**Univariable Analysis**		**Multivariable Analysis**	
	**HR (95%CI)**	**P value**	**HR (95%CI)**	**P value**
Age ≥65 *vs*. <65 y	0.89 (0.51-1.55)	0.67	0.71 (0.37-1.36)	0.31
Male *vs*. female	1.03 (0.59-1.80)	0.92	1.03 (0.55-1.91)	0.94
Current or former *vs*. never smoker	0.70 (0.36-1.36)	0.29	0.78 (0.37-1.64)	0.51
TMB≥median *vs*. <median	0.49 (0.28-0.87)	0.02	0.59 (0.28-1.22)	0.16
PD-L1 status ≥1% *vs*. <1%	0.86 (0.47-1.59)	0.63	1.04 (0.52-2.07)	0.92
*PTPRD/PTPRT* mutant *vs*. wild	0.47 (0.24-0.92)	0.03	0.59 (0.24-1.47)	0.25
**Rizvi2018**				
**Parameter**	**Univariable Analysis**		**Multivariable Analysis**	
	**HR (95%CI)**	**P value**	**HR (95%CI)**	**P value**
Age ≥65 *vs*. <65 y	1.14 (0.86-1.52)	0.35	1.12 (0.69-1.81)	0.66
Male *vs*. female	1.09 (0.83-1.44)	0.54	0.88 (0.53-1.44)	0.60
Current or former *vs*. never smoker	0.69 (0.49-0.97)	0.03	0.59 (0.30-1.17)	0.13
TMB≥median *vs*. <median	0.65 (0.49-0.86)	0.003	0.94 (0.52-1.69)	0.84
PD-L1 status ≥1% *vs*. <1%	0.58 (0.36-0.92)	0.02	0.52 (0.31-0.88)	0.01
*PTPRD/PTPRT* mutant *vs*. wild	0.64 (0.44-0.92)	0.02	0.43 (0.20-0.92)	0.03
Lines of therapy ≥3 *vs*. <3	1.37 (1.00-1.88)	0.05	1.25 (0.70-2.20)	0.45
**All NSCLC in pooled cohort**
**Parameter**	**Univariable Analysis**		**Multivariable Analysis**	
	**HR (95%CI)**	**P value**	**HR (95%CI)**	**P value**
Age ≥65 *vs*. <65 y	1.06 (0.83-1.35)	0.64	0.95 (0.67-1.35)	0.78
Male *vs*. female	1.13 (0.89-1.44)	0.31	1.00 (0.71-1.41)	0.99
Current or former *vs*. never smoker	0.70 (0.52-0.93)	0.01	0.63 (0.41-0.97)	0.04
TMB≥median *vs*. <median	0.56 (0.44-0.72)	<0.001	0.62 (0.42-0.92)	0.02
PD-L1 status ≥1% *vs*. <1%	0.60 (0.43-0.84)	0.003	0.67 (0.47-0.96)	0.03
*PTPRD/PTPRT* mutant *vs*. wild	0.54 (0.39-0.73)	<0.001	0.52 (0.31-0.87)	0.01
lines of therapy ≥3 *vs*. <3	0.88 (0.69-1.12)	0.30	0.80 (0.56-1.13)	0.21

### Association Between *PTPRD*/*PTPRT* Mutation and Better OS Benefit in NSCLC Patients Who Received ICBs Therapy

**Figure 4:**
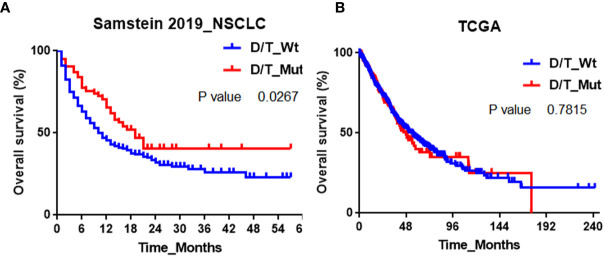
Association between *PTPRD/PTPRT* mutation and OS in **(A)** Samstein2019 and **(B)** TCGA cohorts.

To explore whether *PTPRD/PTPRT* mutation is a predictive or prognostic biomarker for NSCLC, we retrieved the OS data from the Samstein2019 cohort and the Cancer Genome Atlas (TCGA) respectively. Samstein2019 cohort contains 350 advanced NSCLC patients treated with anti-PD- (L)1 monotherapy or combined therapy, and their tumor tissues were profiled with MSK-IMPACT gene panels (341-gene or 410-gene panel). Forty-three (12%) patients harbored *PTPRD* mutation and thirty-five (10%) patients harbored *PTPRT* mutation. Eleven (3%) patients carried both *PTPRD* and *PTPRT* mutation. The overall frequency of *PTPRD/PTPRT* mutation was 19% (**Table S1**). Noted that 206 patients in Samstein2019 cohort were also contained in the Rizvi2018 cohort. **Figure 4A** showed that a significant OS benefit was observed in NSCLC patients with *PTPRD/PTPRT* mutant-type compared to that with *PTPRD/PTPRT* wild-type in Samstein2019 cohort. The median OS in *PTPRD/PTPRT* mutant-type *vs*. wild-type NSCLC patients were 19 months *vs*. 10 months (P=0.03). As for *PTPRD* alone, the median OS in *PTPRD* mutant-type *vs*. wild-type NSCLC patients were 21 months *vs*. 11 months (P=0.01, **Figure S1A**). As for *PTPRT* alone, the median OS in *PTPRT* mutant-type *vs*. wild-type NSCLC patients were 19 months *vs*. 11 months (P=0.17, **Figure S1B**). The results of univariable and multivariable analysis in Samstein2019 cohort were summarized in **Table S2**. *PTPRD/PTPRT* mutation was significantly associated with better OS benefits in the univariable analysis (HR 0.66, 95% CI, 0.45-0.96, P =0.03) and in the multivariable analysis (HR 0.52, 95% CI, 0.31-0.87, P =0.045).

In contrast, for NSCLC patients who do not receive ICBs treatment (TCGA cohort), PTPRD/PTPRT mutation was not associated with a better OS benefit (**Figure 4B** **)**. In TCGA, the *PTPRD* or *PTPRT* incidences are 13.7% and 10.7% in NSCLC respectively. The median OS in *PTPRD/PTPRT* mutant-type *vs*. wild-type NSCLC patients were 47.4 months *vs*. 53.3 months (P=0.78). As for *PTPRD*, the median OS in *PTPRD* mutant-type *vs*. wild-type NSCLC patients were 50.5 months *vs*. 54.1 months (P=0.67, **Figure S1C**). As for *PTPRT*, the median OS in *PTPRT* mutant-type *vs*. *PTPRT* wild-type NSCLC patients were 38.9 months *vs*. 54.1 months (P=0.22, **Figure S1D**). These results suggested that *PTPRD/PTPRT* mutation was a potential positive predictor for clinical benefit of anti-PD-(L)1 therapy in NSCLC instead of a prognosis factor for NSCLC.

### Impact of *PTPRD/PTPRT* Mutation on TMB or Immune-Related Gene Signatures

**Figure 5:**
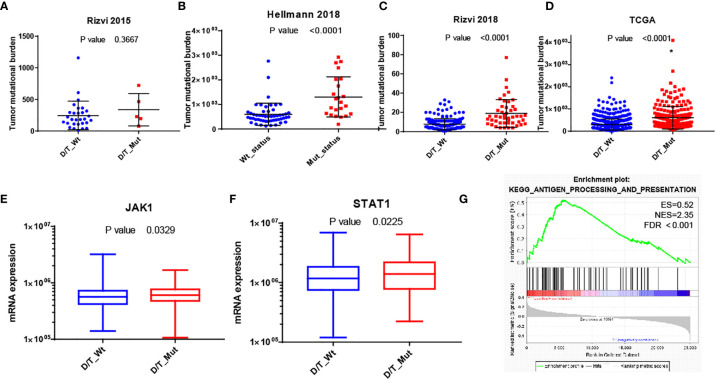
Possible mechanism of the association of *PTPRD/PTPRT* mutation and better clinical outcomes of ICBs therapy. **(A–D)** Comparison of tumor mutational burden between *PTPRD/PTPRT* mutant-type and wild-type NSCLC patients in Rizvi2015, Hellmann2018, Rizvi2018 and TCGA cohorts respectively. **(E, F)** Comparing of mRNA expression of JAK1 and STAT1 between *PTPRD/PTPRT* mutant-type and wild-type NSCLC patients. **(G)** GSEA reveals prominent enrichment of signatures related to antigen processing and presentation in NSCLC patients with PTPRD/PTPRT mutation. *PTPRD/PTPRT* mutation: D/T_Mut; *PTPRD/PTPRT* wild-type: D/T_Wt.

To further understand the underlying mechanism of the association between *PTPRD/PTPRT* mutation and better clinical outcomes in NSCLC patients who received ICBs therapy, the impact of *PTPRD/PTPRT* mutation on TMB or immune-related gene signatures was explored. **Figures 5A–D** demonstrated that *PTPRD/PTPRT* mutation was associated with higher TMB in Rizvi2015, Hellmann2018, Rizvi2018 and TCGA cohorts (P<0.0001). We then analyzed the mRNA data from TCGA to compare mRNA expression of immune related genes between *PTPRD/PTPRT* mutant-type and wild-type NSCLC patients. The list of immune-related genes in **Table S3** was analyzed in this work. As shown in **Figure S2** and **Figures 5E, F**, the mRNA expressions of JAK1 and STAT1 were higher in *PTPRD/PTPRT* mutant-type than wild-type NSCLC patients (p < 0.05). What’s more, GSEA was performed on gene sets in the Kyoto Encyclopedia of Genes and Genomes (KEGG) database. **Figure 5G** revealed enrichment of genes involved in antigen processing and presentation pathways were significantly enriched in NSCLC with *PTPRD/PTPRT* mutation (NES=2.35; FDR< 0.001).

### Mutational Profiles of *PTPRD* or *PTPRT* in Chinese NSCLC 3DMed Cohort

To investigate the mutational profiles of *PTPRD* or *PTPRT* in Chinese NSCLC population, a total of 1224 cases of Chinese NSCLC who have undergone 733 cancer gene-panel *via* next-generation sequencing (NGS) were included in this study, including 886 patients with lung adenocarcinoma and 188 patients with lung squamous cell carcinoma (**Table S4**). There were 768 male and 456 female patients. The median age was 63 (range 22-91). A total of 112 (9.2%) NSCLC patients with *PTPRD/PTPRT* mutation were identified. The incidences of patients with *PTPRD* or *PTPRT* mutation were 5.4% (N=66) and 4.7% (N=57) respectively, which was significantly lower than that of TCGA cohort (*PTPRD*: 5.4% *vs* 13.7%, p <0.0001; *PTPRT*: 4.7% *vs* 10.7%, p <0.0001). Such difference might result from the population. **Figure S3** described the graphical distribution of *PTPRD* and *PTPRT* mutation sites in Chinese NSCLC patients. No clear hotspot mutations and mutated codons were spread throughout *PTPRD* and *PTPRT*, including the phosphatase and extracellular domains, which was consistent with previous work (20).

## Discussion

In this work, *PTPRD/PTPRT* mutation was firstly identified as positive factor for better clinical benefit in NSCLC patients who received ICBs treatment. While no clinical benefits of OS were achieved in patients who do not receive ICBs treatment (TCGA cohort). Moreover, univariable and multivariable analysis further confirmed that *PTPRD/PTPRT* mutation was an independent positive predictor in pooled cohort. In the exploratory analysis, higher TMB and increased expression of genes related to JAK-STAT pathway activation served as potential mechanism underlying the predictive value of *PTPRD/PTPRT* mutation in NSCLC population. GSEA also revealed prominent enrichment of signatures related to antigen processing and presentation in patients with *PTPRD/PTPRT* mutation. Such results suggested that *PTPRD/PTPRT* mutation might be a potential positive predictor of NSCLC patients treated with ICBs.

Protein tyrosine phosphorylation is an important signaling event involved in a wide range of physiological processes in tumor development, whose level is balanced by antagonistic activities of protein tyrosine kinases and protein tyrosine phosphatases (PTPs) (21, 22). The classical PTPs are usually divided into two large groups according to their overall structure, including cytoplasmic non-receptor-type PTPs (NR-PTPs) and transmembrane R-PTPs. Noted that R-PTPs contain PTP domains and extracellular domains, which have intrinsic ability to transduce signals across the cell membrane (23). R-PTPs could not only antagonize tyrosine kinases but also engage extracellular ligands (24). Among R-PTPs, *PTPRD* and *PTPRT* belong to type IIa and IIb R-PTPs, respectively. *PTPRD* or *PTPRT* are identified as tumor suppressor, which is frequently inactivated and mutated in various human cancers. (25–30) For example, the reduced expression of *PTPRD* correlated with poor prognosis in gastric adenocarcinoma (31). Hsu et al. reported that deleterious mutations of *PTPRT* and *PTPRD* was significantly associated with bevacizumab resistance in metastatic colorectal cancer patients (16). Another study suggested that missense mutations in the catalytic domain of *PTPRT* or *PTPRD* were implicated in reducing its phosphatase activity, and mutations in the extracellular domain impair its function in cell adhesion (32, 33). Recently, Li et al. analyzed 2129 pan-cancer patients treated with ICBs, whose cancer genomic data are from the cBioPortal database (34). Compared with *PTPRT* wild-type, *PTPRT* nonsynonymous mutations were associated with better OS in melanoma (N=596) and in pan-cancer (N=2129), and was associated with better PFS in NSCLC (N=510). In the present work, we supposed that the association between *PTPRD/PTPRT* nonsynonymous mutation and good clinical outcomes of ICBs in NSCLC may partially on account of the up-regulation of JAK1 and STAT1 mRNA expression, which subsequently control the expression of chemokines with potent chemoattractant effect on T cells.

To our best knowledge, this is the first work to explore the relationship between *PTPRD/PTPRT* mutation and ICBs treatment in NSCLC patients. In view of the intrinsic property of retrospective study, several limitations exist in the present work. This analysis was based on public cohorts of NSCLC patients who underwent WES or multi-gene panel sequencing, which may yield selection bias. The possible mechanism of the association of *PTPRD/PTPRT* mutation and clinical outcomes of ICBs was performed in TCGA cohort. The application of these conclusions in the present study might be restricted by the limited quantity of patients. Such results should be confirmed in large cohorts.

## Conclusion

Taken together, our results suggested that, in NSCLC patients receiving ICBs, *PTPRD/PTPRT* mutation was associated with better PFS and OS by increasing TMB and immune-related gene signatures. *PTPRD/PTPRT* mutation might be an important component of the immunogenetic landscape and should be integrated into predictive biomarker panels for ICBs therapy. In view of the intrinsic property of retrospective study, such conclusions should be validated in future prospective clinical trials.

## Data Availability Statement

The datasets presented in this study can be found in online repositories. The names of the repository/repositories and accession number(s) can be found in the article/**Supplementary Material**.

## Ethics Statement

All human sample collection and usage were in accordance with the principles of the Declaration of Helsinki and approved by Affiliated Hospital of Nantong University. This study was approved by the Ethics Committee of Affiliated Hospital of Nantong University(2019-K065). All participated patients provided written consents.

## Author Contributions

HK, ML, XW, and BW designed and performed the experiments, prepared the figures and decided to publish. JZ, FT, and BX collected clinical data. ZY and GW analyzed the clinical and TCGA data. ZY and SC prepared the manuscript. All authors contributed to the article and approved the submitted version.

## Conflict of Interest

ZY, GW and SC were employed by 3D Medicines Inc.

The remaining authors declare that the research was conducted in the absence of any commercial or financial relationships that could be construed as a potential conflict of interest.

## Supplementary Material

Supplementary Figure 1 Association between **(A)** *PTPRD* or **(B)** *PTPRT* mutation and OS in Samstein 2019 cohort respectively. Association between **(C)** *PTPRD* or **(D)** *PTPRT* mutation and OS in TCGA cohort respectively.

Supplementary Figure 2 Heatmap depicting the mRNA expression of immune-related genes between *PTPRD/PTPRT* mutant-type and wild-type NSCLC patients.

Supplementary Figure 3 Graphical distribution of **(A)** *PTPRD* and **(B)** *PTPRT* mutations in Chinese 3Dmed_NSCLC cohort.

Supplementary Table 1 Baseline characteristics of Samstein 2019.

Supplementary Table 2 Univariable and multivariable analysis of OS in Samstein 2019.

Supplementary Table 3 The list of immune-related genes analyzed in the work.

Supplementary Table 4 Baseline characteristics of 3DMed NSCLC cohort.
