# Supplementary figures and images for: Association of PTPRD/PTPRT Mutation With Better Clinical Outcomes in NSCLC Patients Treated With Immune Checkpoint Blockades
Source: Front Oncol. 2021 May 27;11:650122. doi: 10.3389/fonc.2021.650122 (PMC8192300; doi:10.3389/fonc.2021.650122)

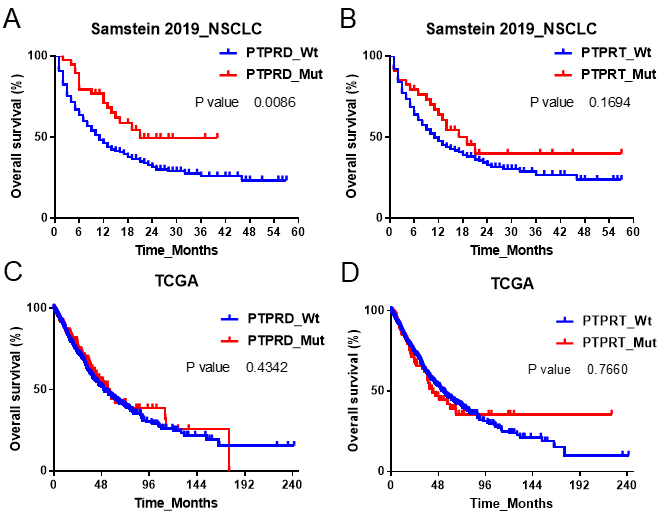

Supplement: Supplementary Figure 1 — Association between (A) PTPRD or (B) PTPRT mutation and OS in Samstein 2019 cohort respectively. Association between (C) PTPRD or (D) PTPRT mutation and OS in TCGA cohort respectively. [file Image_1.jpeg]

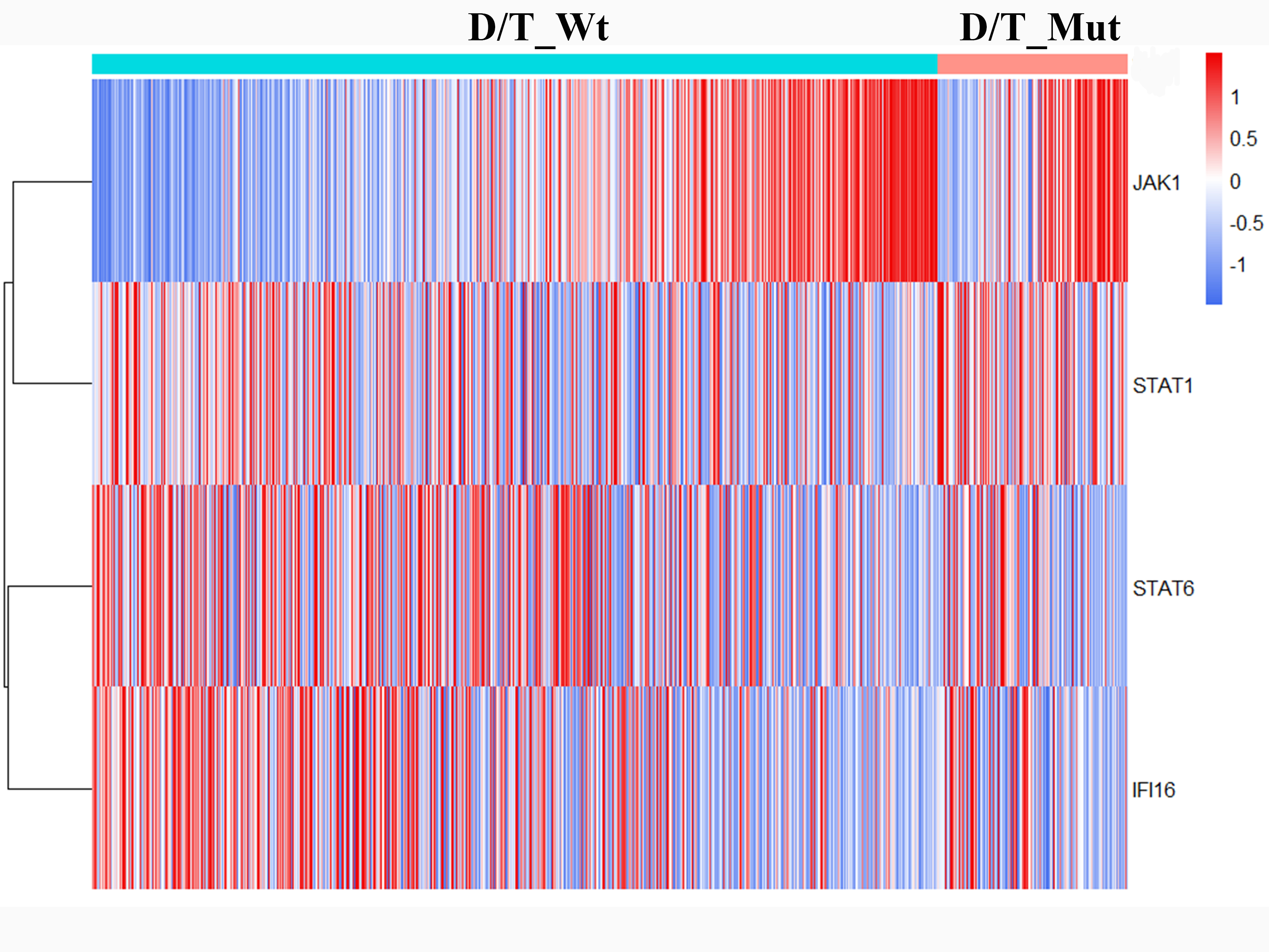

Supplement: Supplementary Figure 2 — Heatmap depicting the mRNA expression of immune-related genes between PTPRD/PTPRT mutant-type and wild-type NSCLC patients. [file Image_2.jpeg]

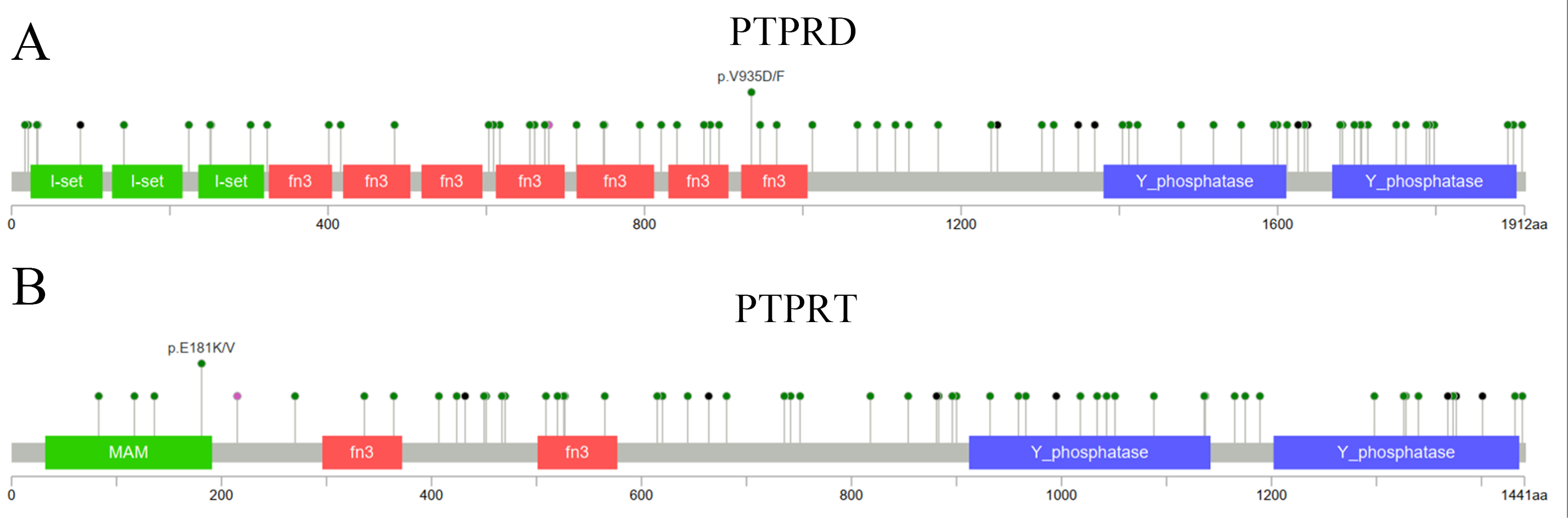

Supplement: Supplementary Figure 3 — Graphical distribution of (A) PTPRD and (B) PTPRT mutations in Chinese 3Dmed_NSCLC cohort. [file Image_3.jpeg]
