# Supplementary material for: Association of PTPRD/PTPRT Mutation With Better Clinical Outcomes in NSCLC Patients Treated With Immune Checkpoint Blockades
Source: Front Oncol. 2021 May 27;11:650122. doi: 10.3389/fonc.2021.650122 (PMC8192300; doi:10.3389/fonc.2021.650122)
Supplement: Supplementary Table 1 — Baseline characteristics of Samstein 2019. [file Table_1.docx]

**Table S1**. Baseline characteristics of Samstein 2019.

| **Characteristics** | **Samstein 2019_NSCLC** |
| --- | --- |
| Total n | 350 |
| Age, median (range) | 67 (31-90) |
| Sex |  |
| Male | 170 (49%) |
| Female | 180 (51%) |
| Cancer type n (%) |  |
| Adenocarcinoma | 271 (77%) |
| Squamous | 45 (13%) |
| Others | 34 (10%) |
| Agent |  |
| PD-(L)1 | 21 (6%) |
| PD-(L)1+CTLA-4 | 329 (94%) |
| Gene, n (%) |  |
| *PTPRD* mutation | 43 (12%) |
| *PTPRT* mutation | 35 (10%) |
| *PTPRD/PTPRT* mutation | 67 (19%) |
| *PTPRD/PTPRT* wild-type | 283(81%) |
