# Supplementary material for: Association of PTPRD/PTPRT Mutation With Better Clinical Outcomes in NSCLC Patients Treated With Immune Checkpoint Blockades
Source: Front Oncol. 2021 May 27;11:650122. doi: 10.3389/fonc.2021.650122 (PMC8192300; doi:10.3389/fonc.2021.650122)
Supplement: Supplementary Table 2 — Univariable and multivariable analysis of OS in Samstein 2019. [file Table_2.docx]

**Table S2** Univariable and multivariable analysis of OS in Samstein 2019.

| **Samstein 2019 cohort** | | | | | | | |
| --- | --- | --- | --- | --- | --- | --- | --- |
| **Parameter** |  | **Univariable Analysis** | |  | **Multivariable Analysis** | |  |
|  |  | **HR (95%CI)** | **P value** |  | **HR (95%CI)** | **P value** |  |
| Age ≥65 vs <65 y |  | 1.12 (0.843-1.476) | 0.44 |  | 1.09 (0.822-1.444) | 0.55 |  |
| Male vs female |  | 1.14 (0.87-1.50) | 0.34 |  | 1.15 (0.87-1.51) | 0.32 |  |
| TMB≥median vs <median |  | 0.86 (0.66-1.14) | 0.29 |  | 0.62 (0.42-0.92) | 0.97 |  |
| *PTPRD/PTPRT* mutation vs wild |  | 0.66 (0.45-0.96) | 0.03 |  | 0.52 (0.31-0.87) | 0.045 |  |
