# Supplementary material for: Association of PTPRD/PTPRT Mutation With Better Clinical Outcomes in NSCLC Patients Treated With Immune Checkpoint Blockades
Source: Front Oncol. 2021 May 27;11:650122. doi: 10.3389/fonc.2021.650122 (PMC8192300; doi:10.3389/fonc.2021.650122)
Supplement: Supplementary Table 3 — The list of immune-related genes analyzed in the work. [file Table_3.docx]

**Table S3.** The list of immune-related genes analyzed in the work.

| B2M | CD137 | CD27 | CD274 | CD28 | CD4 | CD40 | CD80 | CD86 |
| --- | --- | --- | --- | --- | --- | --- | --- | --- |
| CD8A | CD8B | CTLA4 | CXCL10 | CXCL9 | CXCL11 | EOMES | GZMA | IFI16 |
| IFI30 | IFNG | IL15RA | IRF1 | JAK1 | JAK2 | JAK3 | STAT1 | STAT2 |
| STAT3 | STAT4 | STAT5A | STAT5B | STAT6 | TYK2 | OX40 |  |  |
