# Supplementary material for: Association of PTPRD/PTPRT Mutation With Better Clinical Outcomes in NSCLC Patients Treated With Immune Checkpoint Blockades
Source: Front Oncol. 2021 May 27;11:650122. doi: 10.3389/fonc.2021.650122 (PMC8192300; doi:10.3389/fonc.2021.650122)
Supplement: Supplementary Table 4 — Baseline characteristics of 3DMed NSCLC cohort. [file Table_4.docx]

**Table S4.** Baseline characteristics of 3DMed NSCLC cohort.

| **Characteristics** | **3Dmed_NSCLC** |
| --- | --- |
| Total n | 1224 |
| Age, median (range) | 63 (22-91) |
| Sex |  |
| Male | 768 (63%) |
| Female | 456 (37%) |
| Cancer type n (%) |  |
| Non-squamous | 886 (72%) |
| Squamous | 188 (15%) |
| Others | 150 (12%) |
| Gene, n (%) |  |
| *PTPRD* mutation | 66 (5%) |
| *PTPRT* mutation | 57 (5%) |
| *PTPRD/PTPRT* mutation | 112 (9%) |
| *PTPRD/PTPRT* wild-type | 1112 (91%) |
